# The Calgary Audit and Feedback Framework: a practical, evidence-informed approach for the design and implementation of socially constructed learning interventions using audit and group feedback

**DOI:** 10.1186/s13012-018-0829-3

**Published:** 2018-10-30

**Authors:** Lara J. Cooke, Diane Duncan, Laura Rivera, Shawn K. Dowling, Christopher Symonds, Heather Armson

**Affiliations:** 1 Department of Clinical Neurosciences, Cumming School of Medicine, University of Calgary, UCMC Area 3, 3350 Hospital Drive NW, Calgary, AB T2N 4N1 Canada; 2 Cumming School of Medicine, University of Calgary, 3330 Hospital Dr NW, Calgary, AB T2N 4N1 Canada; 3 Physician Learning Program, Cumming School of Medicine, University of Calgary, HSC G302, 3330 Hospital Dr. NW, Calgary, AB T2N 4N1 Canada; 4 Division of Endocrinology & Metabolism, Cumming School of Medicine, University of Calgary, 3330 Hospital Dr NW, Calgary, AB T2N 4N1 Canada; 5 Department of Family Medicine, Cumming School of Medicine, University of Calgary, 3330 Hospital Dr NW, Calgary, AB T2N 4N1 Canada

**Keywords:** Audit and feedback, Feedback, Social learning theory, Framework, Practice improvement, Professional development, Comparative case study, Physician learning, Implementation, Knowledge translation

## Abstract

**Background:**

Audit and feedback interventions may be strengthened using social interaction. The Calgary office of the Alberta Physician Learning Program (CPLP) developed a process for audit and *group* feedback for physicians. This paper extends previous work in which we developed a conceptual model of physician responses to audit and group feedback based on a qualitative analysis of six audit and group feedback sessions. The present study explored the mediating factors for successfully engaging physician groups in change planning through audit and group feedback.

**Methods:**

To understand why some groups were more interactive than others, we completed a comparative case analysis of the six audit and group feedback projects from the prior study. We used framework analysis to build the case studies, triangulated our observations across data sources to validate findings, compared the case studies for similarities and differences that influenced social interaction (mediating factors), and thematically categorized mediating factors into an organizing framework.

**Results:**

Mediating factors for socially interactive AGFS were a pre-existing relationship between the program team and the physician group, projects addressing important, actionable questions, easily interpretable data visualization in the reports, and facilitation of the groups that included reflective questioning. When these factors were in place (cases 1, 2A, 3), the audit and group feedback sessions were dynamic, with physicians sharing and comparing practices, and raising change cues (such as declaring commitments to de-prescribing, planning educational interventions, and improving documentation). In cases 2C–D, the mediating factors were less well established and in these cases, the sessions showed little physician reflection or change planning. We organized the mediating factors into a framework linking the factors for successful sessions to the conceptual model of physician behaviors which these mediating factors drive.

**Conclusions:**

We propose the Calgary Audit and Feedback Framework as a practical tool to help foster socially constructed learning in audit and group feedback sessions. Ensuring that the four factors, relationship, question choice, data visualization, and facilitation, are considered for design and implementation of audit and group feedback will help physicians move from reactions to their data towards planning for change.

## Background

Audit and feedback (AF) is a widely published method of providing performance data to physicians to help them translate knowledge into practice [1]. It has been shown to be more effective in helping physicians change their behavior than many traditional models of professional development [2]. However, the effectiveness of published AF interventions is variable [1]. Several authors have called attention to this issue, citing the need for further study so that the reasons for varied effectiveness of AF can be more fully understood and addressed [3–7].

Here, we extend our previous work, which examined physician responses to a novel type of audit and *group* feedback (AGF) and presented a conceptual model of physician responses to AGF sessions (AGFS) [8]. We observed that physicians engaged in planning for change more robustly in some AGFS than in others. The present study explores factors that influenced the social interactions in those AGFS.

Our understanding of what influences AF effectiveness is informed by three main areas of literature: implementation science, motivational and behavior change theory, and the educational feedback literature [5]. Colquhoun et al. have emphasized the need to draw from these different domains in order to optimize AF design and implementation [5].

Frameworks and theories from these fields can help us to understand the factors that influence implementation [9–19]. One widely cited, evidence-based framework is iPARIHS [17], which identifies four key domains that can be used to determine why an intervention may or may not be successful: the innovation, the recipient, the context, and the facilitation [17].

The authors of iPARIHS described facilitation as the “active ingredient” for implementation [17]. It was defined as “the construct that activates implementation through assessing and responding to characteristics of the innovation and the recipients” in context ([17], p 8). iPARIHS situates the success of implementation upon whether the facilitator can enable the recipients to make the desired change [17].

Because many published AF interventions describe passive, non-facilitated feedback in the form of physician ‘report cards’, the lack of attention to facilitation of feedback in AF is a potential criticism and may explain some of the variation in AF interventions [1, 4, 5, 7].

Brehaut et al. published 15 recommendations to enhance effectiveness of “practice feedback” [7]. Those relevant to the current study include choosing the right items on which to provide feedback (aligned with local priorities, actionable, specific), providing individualized data with relevant comparators, integrating summary messages and data visualization, using social interaction to construct feedback, managing cognitive load, and addressing barriers to change [7].

The use of social interaction to construct feedback underpins the design of AGFS described in the present study and stems from Social Learning Theory [20] and the work of Vygotsky and Ajzen, who emphasized that efficient learning can occur through observation of others’ behaviors, the rewards or consequences of others’ behaviors, and the social norms that develop in group settings [7, 20–22].

Similarly, the Theoretical Domains Framework (TDF) explores factors that impact behaviors [23]. The TDF identifies 14 domains to consider in implementation [23]. These include knowledge and skills, beliefs about capability for change, goal setting, the environmental context and exploration of social influences on implementation [23].

The medical education literature about feedback uptake highlights several aspects of feedback delivery. Ideally, feedback occurs in an in-person, facilitated, coaching-oriented manner within the setting of a trusting, respectful relationship between the provider and recipient of feedback [24–27].

In the R2C2 model, Sargeant et al. emphasize the primacy of the relationship between feedback providers and recipients [25]. Likewise, the R2C2 model focuses on understanding and accepting feedback prior to coaching for change [25, 28, 29]. Watling et al. highlight the value of the credibility of the feedback provider as perceived by the recipient [24, 26]. In an overview of the educational feedback literature, Telio et al. proposed a construct of “educational alliance,” which may influence feedback uptake [27].

The Calgary office of the Alberta Physician Learning Program (CPLP) delivers AF to groups of physicians. This team has delivered over 30 AF projects on various clinical topics, locally and provincially, addressing practice variation and appropriateness. Some projects engaged physicians more than others and we wished to explore why.

In a previous study, we described our approach to AF: audit and *group* feedback sessions (AGFS) [8]. These AGFS were designed based upon the principles of social learning theory and best practices from the education feedback and implementation science literature [7, 17, 23–27]. In the AGFS, physician groups participated in face-to-face, [25, 27] facilitated group feedback sessions with peers, during which they reviewed reports containing their own individualized performance data (along with anonymized peer comparators) in order to identify opportunities, barriers, and enablers for making change [7, 17, 23, 25–27].

We investigated the behaviors of physicians in AGFS in order to capture their reactions and engagement with the data and how the presence of peers influenced the direction of group discussions [8]. Through a qualitative analysis, we developed a conceptual model of how physicians react and interact in AGFS. Physicians expressed initial reactions to the data (skepticism, interest) and then transitioned through several discrete behaviors: understanding and questioning, justifying and contextualizing the data, and reflection and sharing of practices, before beginning to raise ‘change cues’ and make change plans. Change cues were defined as “turning points in the group discussion, initiated by a brief comment highlighting the importance of a performance gap revealed by the data reports,” and were usually raised by a group participant rather than the facilitator, who was not a group member [8].

Qualitative analysis of the AGFS transcripts showed that the degree of interaction and engagement of the physicians varied between AGFS, as did the groups’ tendency to raise change cues and plan for change [8]. This follow-up study sought to explore the factors mediating social interaction during the six AGFS.

We present the results of a comparative case study of the AGFS described in the previous study [8]. The aim was to understand what factors contribute to the successful engagement of physicians in change planning and to develop a practical, evidence-informed tool to guide AGF design and implementation.

## Methods

Ethics approval for this work was received for each case from the Conjoint Health Research Ethics Board: REB13-0075 (case 1); REB14-0484 (cases 2a, 2b, 2c, 2d); REB13-0459 (case 3).

### Setting

This analysis of the work of the CPLP between 2014 and 2016 took place at the Cumming School of Medicine at the University of Calgary. The CPLP is funded by the provincial medical association, to deliver audit and feedback reports about individual practice performance to Alberta physicians. Most projects are initiated when a physician group (such as a department or clinic) approaches the program with a clinical question. Program staff clarify the question; if it is amenable to AF, an algorithm for data extraction is developed and data is accessed from relevant provincial repositories to create the AF report. Each project has a unique, multi-page audit and feedback report reflecting the amount of data and information needed to answer the physician groups’ questions. Confidential reports for participating physicians contain individual data, anonymous group comparators, and relevant references reflecting best practices.

The project culminates in a facilitated AGFS in which consenting physicians have their AF reports (provided at least 1 week before the AGFS), work as a group with a CPLP facilitator to review them, and identify opportunities, barriers and enablers for change [10, 23]. In this study, across all AGFS, the facilitator was the CPLP medical director, who was *not* a member of any of the physician groups. The sessions were attended by CPLP staff who observed the process and served as project managers for each case from conceptualization to delivery.

**Fig. 1:**
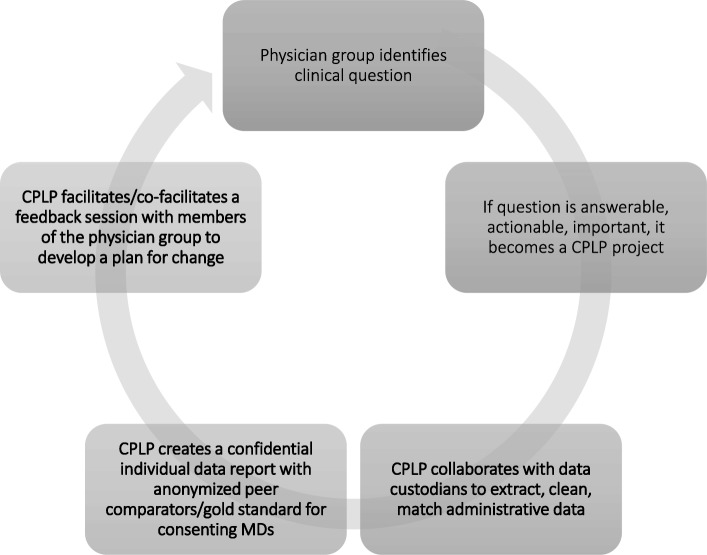
The CPLP process from clinical question to AGFS. Physician groups bring clinical questions of interest for review by the CPLP. The CPLP team reviews the questions for appropriateness for audit and feedback. Consideration is given to impact, reach, actionability, and accessibility of the data. CPLP collaborates with data custodians to make individualized AF reports for consenting doctors. The confidential reports include individual data with anonymous peer comparators and relevant best practice information. Consenting physicians then participate in a facilitated group feedback session with their peers, led by a CPLP and/or participant facilitator. As a group, the physician peers review each aggregate data point, along with their own performance reports and seek opportunities for practice improvement

The process is depicted in Fig. 1.

### Study design

We wished to understand why social interaction between physicians in AGFS varied across cases [8]. Comparative case analysis is an appropriate approach when context, culture, and system factors may influence a program [30, 31]. We used framework analysis to build the individual cases for this research [32–35]. The overall design of the project was as follows: (1) identification of data sources, (2) familiarization with data sources, (3) development of a program model or “change theory,” (4) creation of a framework table from the program model, (5) framework analysis to extract and index data to build individual case studies, (6) comparisons across cases to identify similarities and differences believed to influence social interaction, (7) thematic organization of similar findings into key “mediating factors,” (8) creation of an organizing framework linking the mediating implementation and design factors for social interaction in AGFS to the conceptual model of physician behaviors [8].

#### Participants

Participants included the physicians who participated in the AGFS and the CPLP staff who created the AF reports and delivered the AGFS through collaborative relationships with the physicians. AF participants provided written informed consent to allow access to their administrative health data for purposes of creating AF reports and to record, evaluate, and study the AF sessions in which they participated.

Characteristics of staff who contributed are described in Table 1. The staff worked closely with the physician groups to develop the AGFS and observed the sessions. A typical project would take 1 year to complete. The CPLP staff had extensive longitudinal contact with members of the physician groups and familiarity with processes and contextual and cultural elements that were observed over the course of the projects.

**Table 1:** Descriptions of training and roles of CPLP staff and the research team

CPLP staff participants	Description
Project managers (2)	The two CPLP project managers were experienced in audit and feedback project development and had formal project management training.
CPLP facilitator (CS)	A physician with experience in education and clinical research, who worked at the CPLP for 3 years as the program medical director.
Research team members	
LC	An academic clinician educator with training in medical education research and experience in knowledge translation and audit and feedback, who oversaw the CPLP program at the time when these AGFS occurred.
CS	See above. CS was the medical director at the time the AGFS were completed.
DD	CPLP project and program manager during the time that these AGFS took place. Has training in education and knowledge translation
LR	Research associate in CPLP when the AGFS were conducted. Training in epidemiology, quantitative and qualitative methodologies.
SD	A physician with training in epidemiology and knowledge translation who became the program medical director after the AGFS were developed.
HA	An academic professor of family medicine with training in knowledge translation, medical education and qualitative methodologies with extensive research experience with physician learning and feedback.

Descriptions of the research team are also included in Table 1 in order to acknowledge their orientation, positions, and perspectives at the outset of this research as these perspectives likely inform our analysis.

It is important to note that while CS, LR, DD, and LC participated in the development and evolution of the CPLP processes over time, HA and SD were not involved in the building of the original projects or evolution of the processes described in this study, but rather joined the research team afterwards. Their perspectives were routinely sought in an effort to balance and mitigate any biases or pre-conceived ideas of other team members more intimately involved with the cases.

#### Case definition

For purposes of this study, a “case” was defined as an AGFS with an individual physician group, including the processes of working with the group to refine the question, prepare the report and coordinate, and facilitate the AGFS.

Cases selected for this study included all AGFS that took place through the CPLP between January 2015 and January 2016. These cases were selected because they were offered in short succession, such that researchers could begin to understand the respective cultures, patterns, and influences within each group.

### Data collection

The research team began by identifying possible data sources from which to derive the six case studies. Data sources are listed and described in detail in Table 2. These included sample AF reports from each case, the transcripts and qualitative analysis from the prior study [8], basic data about the projects from a CPLP tracking database (number of reports, dates, ethics approvals, etc.), a process evaluation document that was written for case 1, field notes that were collected directly into our framework table to capture the observations of the research team as they explored the cases, and notes from structured interviews with CPLP staff who were present at the AGFS to corroborate and validate the findings of the research team (detailed later in the methods section).

**Table 2:** Description of data sources for the framework analysis and how they were used by the research team

Data sources	Description of how data was collected/used
Sample anonymous AF reports for each project	The research team reviewed the AF reports and described the quality of data visualization for each case. Through familiarization with the data from the first study, the team captured participant responses to the reports which could support or refute the team observations about the reports. Observations were noted in the framework table (Exemplar graphs from AF reports are shown in Figs. 3 and 4).
Process evaluation for case 1	A formal process evaluation of case 1 was conducted by a senior CPLP team member at the termination of that project. This was a seven-page document outlining processes, procedures, stakeholders, and lessons learned for this project. This report was reviewed by the researchers (LC, DD) information from the report that provided information about influencing social interaction in case 1 was added to the case 1 description in the framework table.
Transcripts and qualitative analysis of AGFS	An inductive thematic analysis of the transcripts for the six AGFS was conducted in a prior study [8]. Team members who reviewed these transcripts repeatedly for the first study made observations about the interactivity, collegiality, and change orientation of the groups which were included in the case analysis. These observations were collected as “field notes” recorded directly into the framework table during research team meetings to discuss the case studies. They were corroborated by returning to review the coding in the transcripts and in interviews with program staff who were present at those AGFS.
Structured interviews of CPLP staff	A staff member who observed each AGFS was interviewed using a the framework table as a structured guide. They were asked to comment about each element in the framework for each case. Their responses were captured in notes entered directly into the framework analysis table. Likewise, the facilitator of the six sessions was interviewed and all responses were captured in the same document.
CPLP tracking document	Basic information about each AGFS was captured by the CPLP staff in a tracking document maintained by the program. These included key performance indicators such as numbers of reports distributed, timing of AGFS.
Observations of the research team	A consensus meeting of members of the research team (LC, HA, LR, DD) was held to share and compare observations of the AGFS and AGF projects. These observations were captured and noted directly into the framework table. This content was reviewed iteratively during the case analysis and during the development of the CAFF to ensure accuracy and consensus about the findings for each case that was analyzed. Observations of the research team were triangulated with the other data sources for corroboration.

Next, the research team built a program model comprised of possible elements influencing the AGF projects [30, 31].

#### Building the program model

Developing a program model is an important early step in a comparative case analysis [30, 31]. The model is comprised of elements that are expected to influence the cases [30, 31]. The research team worked collaboratively over several meetings to diagram the program model based on literature that describe factors that influence implementation success and acceptance of feedback [7, 17, 23–27]. Based on the team’s tacit knowledge derived from their collected experience in developing and delivering audit and feedback and educational feedback as well as their familiarity with the literature, additional elements not specified in published frameworks were added to the program model. The team met, drafted, and re-drafted the model iteratively until there was consensus on the likely factors influencing social interaction in AGFS. This program model, described in the “Results” section, was used as the a priori framework for collecting and organizing information from all of the data sources (Table 3).

**Table 3:** Program model for the comparative case analysis

Program model elements	Components
Innovation [17]	The clinical question upon which the project was based
Style of report [7]	Design of the report, co-creation of report design with CPLP and physician leads from participating groups
Available gold standard/benchmark for the clinical question [7]	
Recipients [17]	Who participants were and how they interacted with one another [7, 17, 23]
Context [17]	Project origin/history Group dynamic (collegiality/culture) and leadership involvement in the sessions [23] Pre-work/relationship building between PLP and participant group [25] Co-creation of data/metrics [7] Proximity of doctor to data/patient (perceived control) [23]
Facilitation [17, 24–26]	CPLP led or co-facilitated with a participant physician-lead Coaching-oriented facilitation [25]
Physician engagement/change talk/change planning [8]	Interactivity of the AGFS, extent of change talk
Outputs	Further project development with CPLP Emergence of autonomous data reporting by the physician group (dashboards) Measured/reported behavior change by the physicians

The program model became the a priori framework into which all data was later indexed in order to build each case study. The framework was put into a table (the “framework table”) with the elements of the framework on the vertical axis and the cases on the horizontal axis.

Three 60-min interviews structured upon the a priori framework comprised one source of data about the cases. The interview participants (the CPLP medical director and two CPLP project managers, detailed in Table 1) were asked to describe their observations of the cases with respect to each of the framework components. Their responses were recorded directly into the framework table. They were used to corroborate and/or add to the observations and findings of the research team. The notes from the interviews were reviewed, condensed, and reviewed again by the research team and summarized in the case analysis table for purposes of the publication.

### Data analysis

Framework analysis was the primary mode of data analysis in this project [32–35]. This is a method of organizing textual data according to an a priori “coding” framework and is commonly used in social sciences research [32–35]. The first step involves familiarization with the data through iterative reading and discussion. Familiarization with the AF reports and the qualitative data from the prior study took place during project development within the CPLP and in conducting the prior study [8]. Next was developing the program model and a priori framework as outlined in the previous section. Data were indexed in the framework table by searching the data sources for evidence for each element of the a priori framework in each case and populating the table with findings. If observations from the data sources did not align with the framework, there was opportunity to add additional elements.

The research team then met to review the case studies and develop consensus on the case descriptions. Corroborating evidence for the observations were sought by triangulating the evidence from different data sources.

The next step in data analysis was the case comparison. Once consensus was reached on the case descriptions, the research team worked collaboratively to identify similarities and differences across the cases. Each of these was considered by the team as to whether or not they influenced the social interactions in the respective AGFS.

The final step in the analysis was to create an organizing framework. The research team iteratively diagramed a framework that captured and organized factors that were identified as likely influences on physician engagement in the AGFS. Similar elements were grouped thematically under a single “mediating factor” for AGFS success. Each mediating factor was then ordered to reflect the CPLP processes and the role these processes might play in the physician behaviors identified in the previous study [8].

## Results

### Participants

A total of 99 AF reports were distributed and 50 physicians participated across the six AGFS. Two CPLP project managers and the CPLP Medical Director were interviewed for data collection.

### Program model

The program model, shown in Table 3, is comprised of eight elements. These elements were derived from two widely cited frameworks that describe influencing factors in implementation [17, 23], Brehaut’s recommendations for enhancing “practice feedback”, and elements from several models of educational feedback [7, 24–27]. In addition to the factors derived from the literature, the team added these elements: AGFS interactivity and change talk and group dynamic.

### Case descriptions

*Case 1* was a project exploring practice variation with specialists involved in a specific surgical procedure. The group demonstrated a collegial relationship during the AGFS with a high frequency of sharing and reflecting on common practices and raising change cues which led to change talk between the participants during the AGF session. Representative examples are listed in Table 4.

**Table 4:** Representative examples of data that were used in building the case studies. In this table, the way in which raw data was indexed to the components of the program model is shown. Where relevant, how this data was linked to the key elements of the CAFF model is also shown in order to demonstrate the way in which inferences were drawn between the original raw data, the program model, and, ultimately, the framework

CAFF model element (overarching theme)	Component of program model	Example findings from case analysis extracted from transcripts	Representative quotations
Facilitation: Facilitator encouraged and created space for these change cues to be raised and discussed by group.	Physician engagement/change talk/change planning [8]	A high degree of social interaction in case 1. Data captured from original AGFS transcripts	Reflecting and sharing of practices around using intravenous anesthesia: “I find that the distinction between deep and awake has become blurred with TIVA. I just turn down the TIVA and just extubate them and they’re almost awake but they’re not as deep as for gas extubation. I usually call it deep but it’s not really a deep extubation in the truest sense… it kind of is halfway in between.” A change cue from another participant: “Our biggest problem is post-op pain” This change cue led to a discussion between the participants about strategies for improving pain management without worsening nausea and vomiting.
Facilitation: Facilitator encouraged and created space for this discussion to occur.	Physician engagement/change talk/change planning [8]	Case 2A Data captured from original AGFS transcripts	A participant expressed their desire to improve their prescribing habits: “…Let us say 3 calls that I get at night are requests, this patient wants a sleeping pill. I might be successful in 1 in 3 in convincing the patient that no they do not really need this. Or convincing the nurse, no they do not really need this. Maybe I’ll be successful with 1 on 3. But its’ worth trying.” This change cue led to a discussion around the need for the group to develop consensus on de-prescribing the compounds in question.
	Recipients and context	Case 2D: The physician group in this hospital was young and they believed their group make-up and relationships with consultants at that site influenced their prescribing habits. Data from original AGFS transcripts	“We are a relatively younger group compared to some... And I think a lot of us try to utilize… restraint in antipsychotic prescriptions. We have had good teams involved with a lot of our patients and we try and avoid a lot of that.”
Data representation	Style of report	Case 2D: Participants raised concerns about the complexity and cognitive load in their AF reports. Data from original AGFS transcripts	“But when you first look at the document… it is very difficult to understand a lot of it, because there’s a lot of information.”
		Examples of data extracted from interviews with CPLP Staff	Representative quotations
Relationship building and question choice	Recipients, innovation, and context	When asked about how the project originated and nature of the innovation (case 1):	CPLP facilitator: “Many of these docs had been involved in [our first] project, but there were some younger recent grads who were more involved in choosing questions—NAME was seen as a strong leader, group had bought into his vision of education in general and the idea of audit and feedback. Maybe the group generally has a culture of monitoring and data collection—… not a big sell to this group – they were keen, thoughtful, interested, and academic in their thinking….they had the advantage of having already been through the process. Lots of groundwork done by our team working with the clinic staff, daycare staff, data analyst, etc... lots of face time from PLP on site—seemed to be helpful with buy in.”
Relationship Building & Question Choice (this group was not directly involved in co-creation of question)	Physician Engagement/Change Talk/Change Planning [8]	When asked about group dynamic (case 2D):	Facilitator: “Poor participation from the group. The group culture at this hospital seemed different from the other groups…very silent during the session…not very many showed up..for their reports”. Project manager 1: “[The participants were] young. Did they feel uncomfortable speaking up?”
Question choice	Innovation	When asked about nature of the project case 3:	Project manager 2: “The goal was hazy…very broad—things that were common”.
Relationship building	Recipients and context	Describing the recipients (case 3):	Facilitator: “This group is super engaged. A bunch of XXX residents who may have been friends before, or became friends afterwards—they are all about the same age, early millenials mostly all on the same page. The group was on the same page. NAME is a competent, quiet leader … very practical, down to earth, that’s why NAME was asked to do this. The group is tight because they cover for one another and cross-cover. The mean age of this group was particularly young – sense of buy in to data, computers, digital natives. Had many suggestions …came up with at least ten potential projects. They were keen – lots of sharing of one another’s data in the sessions”.

Participants in case 1 were familiar with the CPLP approach and staff from a prior project with the program. Their AF reports were co-created with input from the physician lead and three other group members. The physician lead was a highly respected leader in the group. These “group champions” were involved with all aspects of project design from the clinical question to the AGFS. The project lead shared their individual data with the group while co-facilitating the AGFS. This groups’ clinical question related to treatment decisions that were largely under their direct control.

*Cases 2A–D* addressed a de-prescribing question derived from Choosing Wisely Canada recommendations. These cases took place at four adult hospitals, each with separate groups of physicians, leaders, institutional cultures, and serving unique patient populations in different parts of the city. For these reasons, these AGFS are treated as separate cases. These physician groups were reviewing baseline data. While there were varying degrees of change talk and interactivity across these four AGFS, it was uncommon across *all four* AGFS for physicians to compare and share their practices with one another.

*Case 2A* occurred at a hospital serving an area with a large immigrant population. This physician group also provided palliative care. The AGFS focused heavily on discussion about the uniqueness of the patient population. There was skepticism about the data on the basis of the uniqueness of this group’s patient population accounting for the variations in prescribing. The project lead was from this group but was unable to attend this session. The group discussion was interactive and some change cues arose (see Table 4).

*Case 2B* took place in a very large tertiary academic health center serving a complex, high-acuity population. The group was comprised of physicians who worked together for many years. The session was interactive and led to change planning. A nurse with a formal quality improvement role attended this session. A physician participant prompted several interactions around change planning with the allied health staff who shared in-patient care.

*Case 2C* took place in a smaller, community-based hospital serving a predominantly elderly population. One of the physicians for the project was based within this group, attended the session, helped facilitate, and shared their data with the group. This session was less dynamic than cases 2A and 2B. During the AGFS, there were few instances of comparing and sharing practices however, two change cues were raised and led to change talk around improving documentation and discharge summaries as a way to improve community prescribing.

*Case 2D* took place in a newer, smaller hospital on the outskirts of the city. Because of its short history, this hospital has a unique physician culture and medical culture with a focus on patient and community-centered care. Participants expressed that they believed that the make-up of their group and their consultant colleagues influenced their prescribing behaviors (Table 4).

This session was the least interactive of our six cases; physicians did not raise change cues, share their practices, or discuss making changes. The facilitator posed few questions to the group in this session, and few questions were raised beyond trying to understand the data that was presented. Participants pointed out that the AF report was quite difficult to interpret; something that was reflected across cases 2A–D in the amount of time spent questioning the facilitator to understand the data during these AGFS (see Table 4). The facilitator expressed that this group did not seem “interested” in the AGFS.

*Case 3* was a project with a group of specialists addressing broad questions about practice variation in relation to anesthesia for various surgical procedures. In this AGFS, there was a high degree of interactivity around change cues which led to change planning, and also comparing and sharing of practices. Participants raised ideas about additional rounds and education, improvements in charting, and working with other system “actors” to foster improvements in care. The participants were young and very collegial with one another. Several of them had reportedly trained together. The medical lead for the project co-created the questions with the CPLP medical director, helped with report design, pre-circulated relevant journal articles to the participant group, and shared their data with the group during the presentation.

### Comparative case analysis

The results of the comparative case analysis are summarized in Table 5. While the authors recognize that physician behavior change was the goal of undertaking the AGF projects, the focus of this study was the factors influencing whether physicians engaged with the data and change planning during the AGFS. Thus, the “success” of the individual *sessions* was gauged based on the following three criteria: (1) The physicians engaged in the group discussion about the data, (2) Change cues that arose were followed by change talk, and (3) Further action was taken based on the AGFS to address identified gaps. There were numerous additional outputs that arose following the AGFS studied, and these are captured in Table 3 but not elaborated here as they are outside the scope of this paper.

**Table 5:** Summary of results of comparative case analysis for six audit and feedback sessions with different physician groups. The program model elements, which formed the framework table used for data analysis and indexing are on the vertical axis of this table and the cases are listed across the horizontal. The results presented below are summarized, for purposes of the publication, from the detailed notations captured in the original framework tables. The data sources from which the data were extracted are described in Table 1

Program model elements		Case 1	Case 2a	Case 2b	Case 2c	Case 2d	Case 3
Innovation		Review of practice variation and outcomes of anesthesia for procedure x	Review of prescribing practice variation for medications in population x at hospital A	Review of prescribing practice variation for medications in population x at hospital B	Review of prescribing practice variation for medications in population x at hospital C	Review of prescribing practice variation for medications in population x at hospital D	Survey of practice variation in anesthesia for 5 procedures at Hospital A. A survey/overview of practice type of project in preparation for a more subsequent specific project. Intent to introduce the group to A&F
			For this project, A&F was one component of a multifaceted intervention: It was preceded by a didactic inter-professional (doctors, nurses, pharmacists) continuing medical education session, and subsequently included a communication campaign and changes to physician order entry sets, which stemmed from this series of AGFS (cases 2A–D)				
Style of audit report		Highly refined with heavy group input	The reports across cases 2A–D presented the same aggregate data, but had individual physician data for the participating physicians at each site. In each of these four AGFS, a significant amount of time was spent with the participants asking questions to try to understand the data as it was presented				Engaged group lead contributed substantially to report design
			One of the two project leads was from this site and helped to refine the question for the AF reports		One of the two project leads for this work came from this site and helped to co-create and refine the question for the AF reports	2D Participants commented during the AGFS that the report was difficult to interpret during the AGFS	
Gold standard for proposed best practice		None	No, but several existing guidelines and recommendations				No, but existing evidence and guidelines.
Recipients		Specialists at hospital 1. 18 physician reports, 13 physicians attended feedback sessions	Generalist physician group, nurse managers, QI lead at hospitals A, B, C, and D respectively (64 physician reports, 28 physicians attended feedback sessions)				Specialist group, hospital D 17 physician reports, 9 physicians attended feedback session
			Participants in this group identified that their patient population included hospice patients and felt that this influenced prescribing behaviors	Participants in this group cared for very high volume services with high degrees of acuity and complexity	Participants at this site identified that they care for an older patient population. This site had access to a geriatric psychiatry service which may have influenced prescribing patterns. They were positively oriented, cohesive, non-judgmental	Participants at this site self-identified as being “younger” and felt that their prescribing behaviors were influenced heavily by their training and by the local consultants with whom they collaborated	
Context	Project origin	A second project with CPLP for this group	A question raised by city-wide Innovation Committee who invited CPLP to develop the AF reports and deliver AGFS across the 4 sites				CPLP invited by this group after they learned about case 1
	Group dynamic and leadership involvement for the A&F sessions	Experienced, respected senior physician with strong commitment to professional development. Senior physician and 3 colleagues involved in all aspects of project. Cohesive, collegial group. Senior physician and 1 colleague led session and shared own data.	One project lead was a member of this group but was not able to attend this session. The site lead was present but did not have facilitator role in session. Nonetheless, CPLP staff identified that the site lead was “a strong lead” and contributed to the session meaningfully. Lots of questioning and discussion about why this site might be different vs others, skepticism regarding data, requests for data that were not included in original project. CPLP medical director-led session	Site lead present. Lots of questioning of data and discussion about how the data was analyzed. Site lead expressed concern re potential defensiveness of group prior to AGFS. Good attendance. CPLP medical director-led session	One of the two project leads was from this site. Some good discussion in session, but CPLP staff noted that participants began sharing and comparing data as the CPLP team left the room. Good attendance. Project lead shared own data. CPLP medical director-led session	Young physician group in a new hospital working in 2 week shifts with lots of handover. Poor attendance, few questions at session. CPLP medical director-led session	Very motivated, dynamic site project lead with an admin position for education within the group. A young physician cohort, digitally literate, open to self-reflection, keen to discuss change and understand findings. Group cohesion and collegiality high, in part because of call structure. CPLP medical director-led session. Physician project helped prompt group discussion and led discussion around evidence
	Pre-work, relationship-building	This was a second project for this group. Lots of interaction with CPLP team in project development	This was a first project for each of the four hospital groups. There were several meetings with the two project leads (From cases 2A and 2C) and a meeting with the city-wide QI group (at site 2B), but not with group members				First contact was by invitation: CPLP presented to the entire physician group to introduce concept of A&F program. Project lead met frequently with MD group and CPLP team
	Co-creation of data/metrics	A working group with a strong lead and three department members and input from MD group developed metrics and report	This was one part of a larger project. Individual physicians at each site did not choose what question would be addressed; however, the question was brought to the CPLP by the two project leads (From sites 2A and 2C) and was aligned with a provincial initiative to de-prescribe in this population. The project leads helped the CPLP team to determine which data points to build into the reports				Strong leadership from site lead and collaborative development of project between site lead and MD group members
	Proximity of doctor to data and patient	Direct physicians administering orders at bedside	In-direct: Admitting physician was not always attending, many orders were PRN and at discretion of nursing staff. MDs did not feel they had good control over nurse discretion nor order entry system				Direct, as in case 1, and MDs had direct access to database designers for data capture/metrics
Facilitation of Session		Co-led by CPLP and group champion who presented own data to group. Prompting and questions from CPLP and from co-facilitator	CPLP facilitator, who was a physician but not a group member, led each session. Facilitation followed structure of the report, table by table. For case 2C, exemplar data from the site lead’s report was presented but the session was still based on structure of the report. Some degree of input from site lead occurred at sites 2A, B, but not at site C or D. *The facilitation style at site D was different* from the previous sessions. This session was largely a didactic presentation from the facilitator to the group. This was the last of four sessions in this project. The facilitator asked fewer questions than in other groups. When interviewed, the facilitator described the feeling that this group was ‘not as interested’ in the report and that attendance at this session was poor				Led by CPLP facilitator with substantive input from project lead. Prompting questions from both CPLP facilitator and project lead
Engagement/change talk/action planning		Very interactive. Lots of sharing of personal practices and planning around additional change activities	Moderate interaction. Some change cues were raised, leading to plans for change, and some sharing of practices occurred	Moderate. A participant spontaneously raised need for change and this led to considerable discussion of how this could be achieved including engagement of nursing staff and making a collective commitment to de-prescribing	Minimal. There was little change discussion during this AGFS, but participants did share and compare practices to some extent	Minimal. The group asked questions related only to understanding the data. No change cues arose in this AGFS	Very dynamic session with lots of questions on evidence directed to the project lead and discussion between group members
Other outputs		Led to 7 subsequent projects with same specialty in different settings with different innovations and to development of continuous reporting platform for all doctors in this specialty	This AGFS occurred after the education campaign that was a part of the larger initiative. There was no change in prescribing from baseline to this AGFS. However, 1 year after this AGFS, there was a relative reduction in prescribing over all four sites of 39% during study period. Engagement of group in a follow-up project looking at another innovation around insulin prescribing, also in conjunction with a larger, health-authority initiated educational intervention. Changes in order-sets, engagement of multi-disciplinary teams				Specific feedback in follow-up from one group member who changed opioid practice subsequently after learning about practice variation vs other members. Led to follow-up projects with same group looking at other clinical questions and ultimately additional projects in city-wide department (see case 1)

In comparing across cases, the most change talk and planning occurred during cases 1, 2A, and 3. Comparative case analysis highlighted several key elements fostering physician engagement with the data in these groups. These included the pre-existing relationship between the CPLP and the physician group (case 1), the active involvement of a physician leader from within each AGF group (cases 1, 2A, and 3), co-creation (between group members and CPLP) of the clinical questions and AF report designs (cases 1 and 3), perceived control over the behaviors being measured in the AF report (cases 1 and 3), intrinsic group dynamic or cohesiveness (cases 1, 2A, 2B, and 3), and the approach of the facilitator leading the sessions (co-facilitation in cases 1 and 3 and coaching-oriented facilitation with prompts and questions in cases 1, 2A, 2B, and 3).

The physicians’ tendency towards sharing and comparing practices during AGFS were greater in AGFS 1 and 3, which were based on clinical questions about practice variation, than they were in cases 2A–D. The data visualization for the AF reports in cases 2A–D was overly complex. During AGFS 2A–D, a disproportionate amount of time was spent by the participants on understanding the reports. Participants in AGFS 2D commented specifically on this finding. In addition, the extent of interactivity in AGFS 2A-D diminished with each successive case. In reviewing the transcripts, and in interviewing the facilitator and CPLP staff who observed the sessions, it became apparent that the facilitation style changed across those four cases. In cases 2A and 2B, the facilitator asked many questions of the participants, prompting them to consider their data. In contrast, in case 2D, the facilitator asked very few questions and the participants made few inquiries about the data. Indeed the transcriptionist for the AGFS described the AGFS for 2D as a “lecture rather than a focus group” after transcribing the session.

#### Developing an organizing framework

**Fig. 2:**
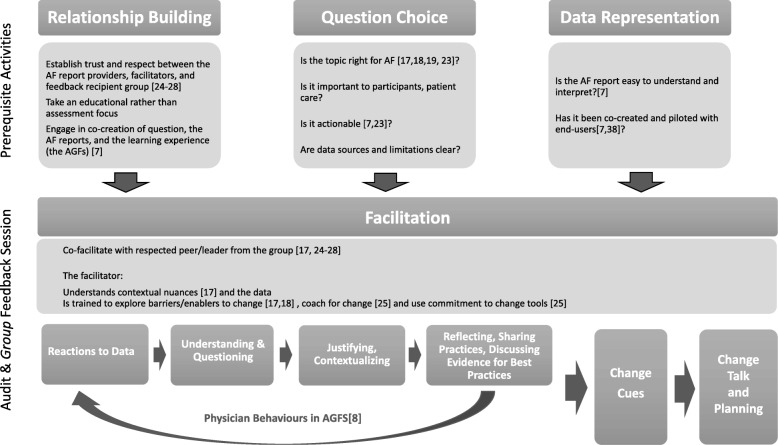
The Calgary Audit and Feedback Framework (CAFF) for the design and delivery of AGF. This model organizes the key findings from our case analysis that were identified as important drivers in moving physicians through the cycle of discrete behaviors that occur in AGFS towards the end goal of planning for change. Under each “mediating factor” are listed the distinguishing elements between the cases with more or less social interaction that emerged from the comparative case analysis. The framework is linked to the conceptual model of physician behaviors in AGFs to show how the different mediating factors drive the behaviors towards change

In reviewing the similarities and differences across the cases, and grouping similar items, four overarching themes emerged: relationship building, question choice, data visualization, and facilitation. In organizing these in a way that reflected both the processes used by the CPLP and the observed physician behaviors in AGFS, it was possible to link the design/implementation factors with the cycle of predictable physician behaviors [8] to create a practical framework for AGF project planning and design. The Calgary Audit and Feedback Framework (CAFF) is shown in Fig. 2.

## Discussion

The audit and feedback literature consists of many published interventions demonstrating that AF can be a useful means to change physician behavior [1]. However, it has been pointed out that there are gaps in the design and implementation of AF and that these may account for some of the variability in the effectiveness of published interventions [1, 3–7].

The CPLP developed a novel way to deliver AF that aimed to address some of the design and implementation elements emerging from the AF and educational feedback literature: AGFS [4–7, 9, 24–27]. Recognizing that social learning was a key ingredient in pivoting AGFS towards change planning, we wished to explore why some AGFS were more socially interactive than others [8, 20].

In this study, we present the results from a comparative case analysis of six AGFS and based on these findings, we propose a practical, evidence-informed framework for the development, and implementation of AGFS. We have termed the product of this research a “framework” based on Nilson’s definition:

“Frameworks in implementation science often have a descriptive purpose by pointing to factors believed or found to influence implementation outcomes.” ([36], p 2).

In the Calgary Audit and Feedback Framework(CAFF), the key findings from the case analyses were grouped into four “factors” and aligned with the conceptual model of physician behaviors to demonstrate linkages between the design and implementation elements and the desired progression of the physicians towards change planning in AGFS (Fig. 2).

### The Calgary Audit and Feedback Framework

The intent in developing the CAFF was to provide a concise way to organize our findings and to delineate how the various design elements could drive physicians towards planning for change in a socially constructed learning environment (Fig. 2). The first two factors of the framework, relationship building and question choice, help physicians in AGFS overcome potential barriers to acceptability of the feedback (for example: skepticism, mistrust, non-actionable feedback). Quality data representation is needed to facilitate understanding and interpretation of the data. Facilitation as a mediating factor is *dependent* on the trusting collaborative relationships between the feedback providers and recipients and helps participants move from understanding the data to change planning. Each of the identified mediating factors are supported by existing literature and can be used to help us understand how social interaction drives physicians towards change planning.

#### Building relationships

Bing-You et al. identified that feedback recipients incorporate feedback into their learning inconsistently [37]. This may be mediated by failure to create an “educational alliance” between the providers and recipients of feedback, the credibility and constructiveness of the feedback, and/or the nature of the relationship and trust between the providers and the feedback recipients [25–28]. To mitigate skepticism and enhance feedback acceptability, the primacy of establishing respect and trust between the “provider” and “recipient” of in-person feedback cannot be over-emphasized [25, 27–29].

We found that when present, skepticism about the data was a barrier to moving physicians in AGFS towards change talk. Groups who had prior experience with the CPLP program had a working relationship with our team and appeared to engage with their data and the facilitator readily.

Key elements of relationship building in our program include empowering a group member to co-facilitate the AGFS, clarifying use of and the confidential nature of the AF reports, and the data limitations. These elements necessitate advance planning and direct contact between the individuals developing the AGF project and the prospective AGF recipients in order to forge an “educational alliance” [27]. We suggest the successful creation of this alliance is reflected in the subsequent engagement of the participating groups in additional projects with the CPLP. Co-creation also appeared to be a critical component in the development of the educational alliance: Cases where co-creation was emphasized in project design had the most interactive AGFS.

#### Question choice

A key contributing factor to intervention effectiveness is ensuring that the intervention selected is appropriate to the desired behavioral change [10, 11, 19]. The Knowledge-to-Action Cycle and the “Behavioural Change Wheel” provide guidance for the choice of intervention for fostering change depending on the nature of the desired behavior change [18, 19].

It follows that not all questions about physician performance are appropriately dealt with by using AF interventions [7, 10, 11, 19, 23]. Choosing metrics over which physicians have little control or for which there is no gold standard is unlikely to result in feedback acceptance [7].

Brehaut and others emphasize the importance of actionability of the feedback provided in AF [7, 10, 19, 26]. Watling et al. found that perceived “constructiveness” of feedback mediates its acceptance by learners to some extent, and we suggest that the perceived “actionability” described in the AF literature [7] and perceived constructiveness of educational feedback are similar constructs [7, 26]. Likewise, the availability of best practice evidence or gold-standards in addition to anonymized peer data with which to compare an individual’s performance may be an important component of the perceived “constructiveness” of the feedback [7, 24, 26].

In AGFS (cases 1 and 3) in which the physicians had direct control over the outcome in question, participants focused readily on the evidence, reflection, and change planning. In contrast, in cases 2A–D, physicians expressed reservations about their ability to make change because of other “actors” in the system who could influence the outcome (e.g., allied health). We observed that this perceived “actionability” seemed to be mediated through the proximity between the physicians’ behavior or decision and the outcome being measured.

#### Representation of the data

**Fig. 3:**
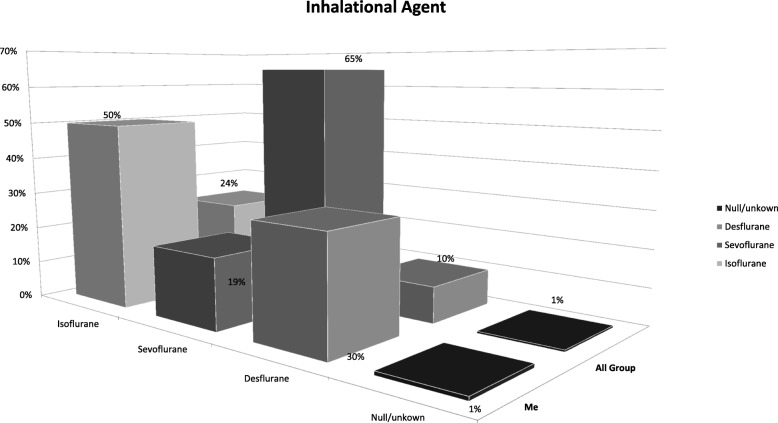
Representative example of a single data table from a generic report from the case 1 project. Physicians appeared to be readily able to interpret these graphs during the AGFS, allowing more time for reflection and planning for change

**Fig. 4:**
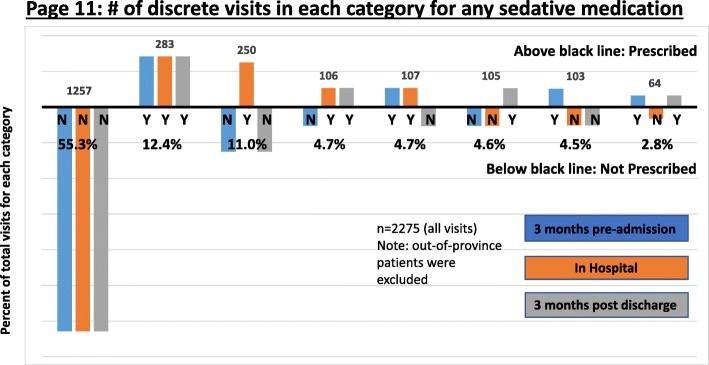
This graph is an aggregate exemplar of how the data for cases 2A–D were initially presented. The goal was to show physicians whether they were discontinuing sedatives and anti-psychotics in patients who were admitted with a pre-existing prescription, whether the patients were started on these medications in hospital, and whether they remained on them after discharge. A desirable outcome for a patient on sedatives or anti-psychotics either before or during their hospital stay was considered to be discharge from hospital without a prescription for these medications. Physicians found these graphs challenging to interpret, resulting in disproportionate AGFS time being spent on clarifying and questioning

How the data is presented in an AF report affects how easily the participants can understand the report and move on to making meaning from the findings and looking for change opportunities. Our analysis revealed that the data reports for cases 1 and 3 were simpler in their design than the others (Figs. 3 and 4); this may be because the project leads were more intimately involved in co-creation of the questions the reports. This highlights the importance of managing cognitive load, building “end-user” testing of the AF reports into the design of an AGF project, and the value of co-creation of the learning [7, 38].

#### Facilitation of the AGF session

The approach to facilitating the AGFS is pivotal to ensure that participants in the session move from interpreting the data to planning for change. The iPARIHS framework identifies the facilitator as playing the central role of fostering interaction between the participant, their data, and their context [17]. The iPARIHS authors emphasize that experienced facilitators can grasp the nuances of system and organizational factors (context) that may influence implementation of a change intervention [17]. The need for an understanding of context lends further support for the role of co-facilitating feedback with a group member. A non-group member facilitator, while expert at interpreting the data risked missing relevant change cues [8]. Physician engagement was highest when a respected group member co-facilitated AGFS, and we speculate that this resulted in positive credibility judgements by the other participants [10, 11, 19, 23, 26]. We suggest that training a member of the recipient group to co-facilitate or lead AGFS will enhance acceptability of the feedback and that with their privileged knowledge (as a group member) of the context and culture of the group, they will be better able to capture change cues and incorporate elements such as barriers and enablers of change to support action planning [17].

Sargeant et al. highlight that the facilitator’s role in a feedback session should be coaching-oriented [25]. The coach-facilitator helps participants to navigate through their reactions to data, to understand their data, and then plan for change [25]. The “coaching-oriented approach,” with prompts and questioning is essential. In our study, when prompts and questioning were not used by the facilitator (perhaps because of facilitator fatigue over the course of four AGFS), there was very limited social interaction in the AGFS [25].

### Limitations and future research

There are several limitations of this study. Our findings of physician behaviors in AGFS represent the collected observations over the course of six AGFS, five hospitals, and three specialties, but included only consenting physicians. The authors acknowledge the risk of selection bias in our participants. Another potential limitation is the perspectives of some of the research team members, who were intimately involved with the leadership of CPLP when this work was carried out. The authors attempted to balance important tacit knowledge that informed the program development with the risk of bias from pre-conceived ideas by acknowledging their perspectives, bringing on experienced research team members who were not intimately involved with the CPLP at the time of the analyses and triangulating across data sources to corroborate our observations. Finally, it may be argued that cases 2A–D should be treated as one case because they all addressed the same clinical question. However, the authors were interested to explore contextual and cultural factors that influenced social interaction in AGFS, and because cases 2A-D occurred in different settings with different participants, it was felt that these case should be treated separately [17, 23].

The framework we present is a synthesis of our findings from analysis of AF projects designed and delivered in a novel way to address recommendations for enhancing AF in the literature [7]. While it appears that design and implementation elements used by the CPLP to deliver AGFS promote social interaction, prospective evaluation and refinement of the CAFF will be important next steps.

## Conclusion

Based on the findings of our study, we present a practical, evidence-informed approach for the design and delivery of AGFS in a way that links design and implementation elements (relationship building, choice of question, quality of data representation, and facilitation style) to the anticipated behaviors of physicians participating in AGFS in order to promote social learning and behavior change.

## Abbreviations

AF: Audit and feedback
AGF: Audit and group feedback
AGFS: Audit and group feedback sessions
CAFF: Calgary Audit and Feedback Framework
CPLP: Calgary Physician Learning Program
